# Understanding Ferruginous Versus Euxinic Conditions by Simulating Microbial Conditions in Meromictic Lakes

**DOI:** 10.1111/gbi.70037

**Published:** 2025-10-20

**Authors:** Vanessa M. Hawkins, Cody S. Sheik, Sergei Katsev

**Affiliations:** ^1^ Department of Physics and Astronomy University of Minnesota Duluth Duluth Minnesota USA; ^2^ Department of Biology University of Minnesota Duluth Duluth Minnesota USA; ^3^ Large Lakes Observatory University of Minnesota Duluth Duluth Minnesota USA

**Keywords:** anaerobic metabolisms, ferruginous lakes, reaction‐transport modeling

## Abstract

Ferruginous (iron‐rich) conditions have been prominent in oceans throughout the Earth's geologic history but now are reliably found only in a handful of permanently stratified lakes. Microbially mediated iron reduction in such anoxic environments competes with sulfate reduction, which promotes euxinic (sulfide‐rich) conditions. Besides the shared demand for organic compounds, the competition is fostered by the produced hydrogen sulfide, which may reduce iron oxides abiotically or co‐precipitate with dissolved iron as iron sulfides. Understanding why some environments develop ferruginous rather than euxinic conditions (or vice versa), as well as the attendant effects on methanogenic fermentation, is key to understanding both modern and ancient anoxic ecosystems. Here, we reproduce biogeochemical distributions in multiple anoxic, low‐sulfate, meromictic lakes around the world using a biomass‐explicit reaction‐transport model with a fixed set of metabolism‐specific microbial parameters. The results suggest that sulfate reduction and methanogenesis are ubiquitous even in iron‐rich systems, and are reflected in microbial surveys. Ferruginous conditions typically develop for surface sulfate concentrations below ≃100 μM. Interestingly, there seems to be a dearth of stably stratified water bodies where sulfate concentrations can persist in the medium‐sulfate range of several hundred μM. Rather, when sulfur burial into the sediments becomes iron limited, sulfate tends to accumulate in the water column to much higher (mM) concentrations. A similar mechanism could be suggested to have operated in the variably sulfidic and ferruginous water columns of early oceans. Model simulations also reveal the previously underappreciated role of physical transport in shaping biogeochemical distributions, as minor variations in mixing rates can lead to large variations in microbial abundances. Model applicability across multiple lakes points to an encouraging possibility that geochemical patterns in complex biogeochemical systems may be described from a small number of thermodynamic and kinetic principles using a minimum of fitting parameters.

## 1 Introduction

Biogeochemical reactions in water and sediment columns of aquatic bodies are catalyzed by functionally diverse microbial populations that compete for substrates and energy resources. Anaerobic mineralization of settling organic matter, in particular, involves several microbial respiratory pathways that often arrange themselves in vertical progressions. Shaped by the outcomes of microbial competitions, these progressions lead to corresponding zonations in water chemistries (Bethke et al. 2011). Anaerobic reduction of nitrate and manganese oxides, being highly energetic and kinetically fast, consumes these oxidants within the topmost anoxic strata. Below, anaerobic metabolisms are dominated by the reduction of the more abundant solid iron (oxyhydr)oxides and dissolved sulfate, and by fermentation, most notably methanogenesis. Geochemical outcomes of these microbially‐catalyzed reactions have shaped the chemistry of the Earth's oceans for most of their geologic history (Lyons et al. 2024). Throughout the Archean and Proterozoic eons and for significant periods in the Phanerozoic (Sperling et al. 2021; Bauer et al. 2022), the global ocean was pervasively anoxic below a shallow thermocline, with deep waters being variably ferruginous (iron‐rich) or euxinic (sulfide‐rich) (Poulton and Canfield 2011; Lyons et al. 2009). Marine concentrations of sulfate played an important role in climate regulation, as sulfate attenuates the upward fluxes of methane via microbially‐assisted anaerobic oxidation, limiting the greenhouse effect (Olson et al. 2016).

Today, with the exception of a few restricted marine basins such as the Black Sea, persistently anoxic water columns are found only in a handful of meromictic lakes. A few of those that have low sulfate concentrations, typically below 100 μM, are ferruginous, with dissolved iron (${\mathrm{Fe}}^{2+}$) accumulating in anoxic strata. Such systems have been extensively studied as modern biogeochemical analogues of the ancient oceans (Swanner et al. 2020; Crowe, Jones, et al. 2008). Applying insights from meromictic lakes to ancient oceans is highly challenging, however, because of the obvious physical differences between the environments. Placing biogeochemical reactions in the appropriate context therefore requires understanding of how their rates, and the activities of the microbial populations that catalyze them, vary with environmental conditions. Such predictive understanding is still very limited, even for modern lakes. It is difficult to say, for example, under what conditions a stratified body of water would be expected to become euxinic, rather than ferruginous. Broad mass‐balance estimates for the oceans suggested that this may happen when the inputs of sulfur (S) into the water body exceed those of the highly reactive iron (${\mathrm{Fe}}_{\mathrm{HR}}$) by a factor of about 1.8 (Poulton and Canfield 2011). Also, mass balance tipping points with respect to the degree of ocean oxygenation have been suggested for the dynamics of the Earth's sulfate reservoir (Fakhraee et al. 2024). Alternatively, an intrinsic bistability related to the formation of iron sulfide minerals was suggested to enable transitions between the iron‐rich and sulfide‐rich states, for example, in response to small changes in organic carbon inputs (van de Velde et al. 2020). Lacustrine estimates, however, have been unavailable to verify such predictions. The extent of sulfate reduction and viability of methanogenesis under ferruginous water column conditions are similarly important (Friese et al. 2021; Bethke et al. 2011). As iron reduction yields more Gibbs free energy per carbon atom than sulfate reduction or methanogenesis, it is often assumed to happen in anoxic waters preferentially. Thermodynamics, however, is not the only factor that determines the priority of redox reactions (Bethke et al. 2011). Non‐thermodynamic factors include, for example, accessibility and crystallinity of mineral surfaces (Roden 2004; Bethke et al. 2011) and pathway‐dependent kinetic capabilities of microbial cells. Physical properties of the environment and the concentrations of substrates also matter: in sediments, the zone of iron reduction almost always overlies the zone of sulfate reduction, but in anoxic water columns with ferruginous deep strata, mid‐water euxinia is the rule.

Addressing these challenges requires developing approaches capable of describing microbial metabolism rates across environments (Jin 2023). Unlike ancient oceans where processes need to be inferred from indirect proxies in the rock record, modern lakes offer a range of conditions for which conceptual approaches can be tested against direct biogeochemical data. At the cell level, a key assumption for cross‐system transferability may be that functionalities of microbial cells are determined by their local microenvironments. When micro parameters (e.g., the pH, temperature, ambient chemical concentrations) are sufficiently specified, one may posit that the growth and metabolic rates of the microbial population may be predictable for catabolically similar microorganisms. This is a non‐trivial assumption, given the taxonomic and metabolic diversity of extant organisms. Cell dormancy (Jin 2023) or microbial adaptations may pose additional challenges by causing cells to manifest different kinetic laws under different conditions (Wu et al. 2022). The assumption is not entirely unreasonable, however, given that microbial growth and function may be evolutionarily optimized for certain conditions that favor specific catabolic pathways. Past biogeochemical models for redox‐stratified aquatic environments have considered microbial populations and growth (Dale et al. 2008; Meile and Scheibe 2019), but parameters describing microbial kinetics have typically been chosen by fitting site‐specific conditions. If cross‐system descriptions are possible under some range of conditions, models should be able to employ consistent sets of parameter values for each functionally defined microbial population.

A recently developed approach (Katsev and Halevy 2025) suggests a way to describe microbial kinetics in terms of experimentally measurable pathway‐specific cell capabilities. The biogeochemical dynamics are then simulated by decoupling the effects of thermodynamic, kinetic, physical, and microbial competition factors. By applying the approach to anaerobic metabolisms in stratified water columns, the study predicted broad patterns of biogeochemical activity for a range of hypothesized marine conditions over the course of the ocean's geologic history. A logical test for this method's validity is applying it to experimentally accessible modern systems using the same set of biogeochemical parameters.

Here we apply the model of Katsev and Halevy (2025) to most of the known ferruginous meromictic lakes and a euxinic meromictic lake. We compare model results against observations, validating the approach, and identify environmental characteristics that lead to the development of ferruginous rather than euxinic monimolimnia. The results help us understand biogeochemical competitions during anaerobic mineralization of organic carbon, reveal broad geomicrobiological patterns in vertically stratified environments, and provide insights into process rates. They also highlight the important and underappreciated role that physical mixing plays in shaping biogeochemical distributions.

### 1.1 The Study Lakes

**FIGURE 1:**
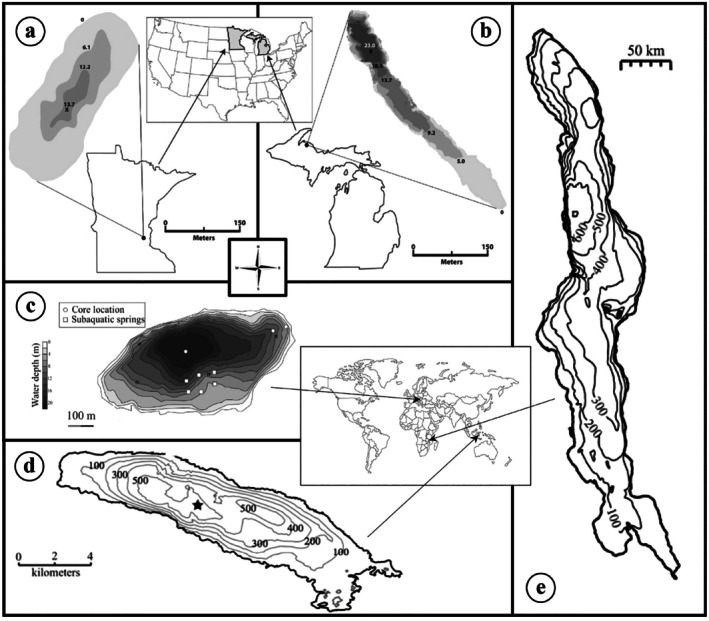
Locations of study lakes and their bathymetries. (a) Brownie Lake, (b) Canyon Lake, (c) Lake Cadagno, (d) Lake Matano, (e) Lake Malawi.

From the known ferruginous meromictic lakes (Swanner et al. 2020; Schultze et al. 2017), we selected those that have sufficient amounts of water column data to allow a reasonable model calibration (Figure 1). We also considered a mildly euxinic meromictic lake to investigate conditions under which transitions from ferruginous to euxinic regimes occur.

*Lake Matano*, a 600‐m deep tropical lake on the Sulawesi island of Indonesia, is arguably the most studied ferruginous meromictic lake. Its stratification likely persisted for at least 600 years (Crowe, O'Neill, et al. 2008). The deep, yet illuminated, chemocline at around 100 m depth hosts a community of photoferrotrophic organisms (Crowe, Jones, et al. 2008). Dissolved iron accumulates in deep monimolimnion to 140 μM, whereas methane concentrations reach 1.4 mM. Important available water column data include sulfate reduction rates (Crowe, Jones, et al. 2008), estimates of downward fluxes of Fe minerals (Bauer et al. 2020), and vertical distributions of turbulent eddy diffusivity $K_{z}$, which characterizes the intensity of diapycnal physical mixing (Katsev et al. 2010). The neighboring 200‐m deep Lake Towuti is also ferruginous (Friese et al. 2021), but with low (μM) concentrations of iron in bottom waters. While it has been also considered meromictic, recent investigations with hydrodynamic and biogeochemical models (Pu et al. 2025) revealed that the lake may have mixed in 2008, with oxygen reaching bottom waters. This would imply that the measured chemical distributions, which were obtained in later years, may not be suitable for calibrating the model, which would need to consider complex temporal dynamics. We therefore exclude Lake Towuti from the present study.

*Brownie Lake* is part of the urban chain of lakes in Minneapolis, Minnesota. It is a small (4 ha, 14 m maximum depth) eutrophic lake that has been affected by anthropogenic activity. A culvert constructed in 1917 decreased the water level, sheltering the lake surface from wind and leading to permanent stratification, which has been exacerbated in recent years by urban road salt runoff (D. C. Smith 2008; Lambrecht et al. 2020). Dissolved $O_{2}$ disappears at the chemocline at a depth of around 5 m (Lambrecht et al. 2018), while dissolved ${\mathrm{Fe}}^{2+}$ reaches 1500 μM near the lake's maximum depth. Methane (${\mathrm{CH}}_{4}$) accumulates to similar concentrations. Sulfate (${{\mathrm{SO}}_{4}}^{2-}$) concentrations in the mixolimnion approach 70 μM (Lambrecht et al. 2020), whereas sulfide ($H_{2}S$) peaks at 75 μM in the chemocline (Wittkop et al. 2020).

*Canyon Lake*, located in the Huron Mountains region of Michigan, is a small (1 ha, 23 m maximum depth) narrow pristine lake. It is naturally meromictic due to protection from wind by the surrounding canyon and groundwater inputs that increase the density of the deep water (Lambrecht et al. 2018; L. Smith 1940). While the thermocline is typically located between 3 and 4 m in depth, oxygen reaches 13–15 m, and a diffuse chemocline extends to 17–19 m (Lambrecht et al. 2018). Concentrations of ${\mathrm{Fe}}^{2+}$ and ${\mathrm{CH}}_{4}$ both reach around 2000 μM. ${{\mathrm{SO}}_{4}}^{2-}$ concentrations in the mixolimnion are around 6 μM (Lambrecht et al. 2020).

*Lake Malawi*, the southernmost of the East African Great lakes (28,800 km^2^, maximum depth 700 m; Katsev et al. 2017), is considered here because it seems to be neither ferruginous nor euxinic, despite its anoxic deep waters. Meromictic stratification is maintained by weak temperature and salinity gradients (Wüest et al. 2019); climate‐driven three‐dimensional circulation renews the deep waters over the decadal time scales. A seasonal thermocline develops at a depth of 30–40 m during the wet season (November to March), and a permanent density gradient is located around 250 m. The water column is anoxic past 200 m. ${{\mathrm{SO}}_{4}}^{2-}$ concentrations are around 17 μM in surface waters and decrease to < 5 μM in the monimolimnion. Hydrogen sulfide ($H_{2}S$) is present in a broad mid‐water layer extending to about 300 m, but at low concentrations (< 5 μM; Li et al. 2018). The deeper waters appear to be free from sulfide. Concentrations of dissolved ${\mathrm{Fe}}^{2+}$ were undetectable (< 2 μM) throughout the water column during the 2013–2014 sampling campaign (Li et al. 2018).

*Lake Cadagno* is a euxinic alpine lake (26 ha, maximum depth 21 m; Gulati et al. 2017) in the Piora valley in southern Switzerland (Del Don et al. 2001). Influx of salt‐rich water from sublacustrine springs stabilizes the lake through crenogenic meromixis. From 1948 to 2007, the lake was dammed and used as a reservoir for a hydroelectric power plant, with stratification persisting through a 3 m decrease in water levels in winter (Bossard et al. 2001; Gulati et al. 2017). A persistent chemocline exists at a depth of 10–13 m (Del Don et al. 2001), and the thermocline is located between 5 and 12 m depth (Bossard et al. 2001). Dissolved $O_{2}$ extends down to 7–10 m. Sublacustrine springs maintain around 350 μM of ${{\mathrm{SO}}_{4}}^{2-}$ at the lake surface and 1500 μM of ${{\mathrm{SO}}_{4}}^{2-}$ in deep monimolimnion, creating an inverted sulfate profile. Concentrations of $H_{2}S$ reach 75 μM (Saini et al. 2022), while concentrations of ${\mathrm{Fe}}^{2+}$ do not exceed 1 μM (Xiong et al. 2019).

## 2 Methods

### 2.1 The Bioenergetic Reaction‐Transport Model

The one‐dimensional biogeochemical reaction‐transport model (Katsev and Halevy 2025) simulates vertical physical transport and the coupled microbially‐catalyzed and abiotic chemical reactions in a stratified water column. The details of the model formulation and the parameters are given in the Supporting Information. Table 1 lists the lake‐specific model parameters. Tables S1 and S2 list the included reactions and kinetic formulations, while the set of parameters commmon for all simulated lakes is given in the Table S3. Briefly, the treatment of microbial pathways broadly follows the energetics approach of González‐Cabaleiro et al. (2016) and Smeaton and Van Cappellen (2018), which is then incorporated into a more traditional reaction‐transport model (e.g., Fakhraee et al. 2017). Cell‐specific kinetics and thermodynamics of microbial metabolisms, and the growth of the corresponding microbial biomass, are simulated for the functionally‐defined microbial groups, such as those performing iron reduction (IR), sulfate reduction (SR), and methanogenesis (MG). Cell growth yields Y per catabolic reaction turnover are calculated from the concentration‐dependent Gibbs free energies of the catabolic and anabolic reactions (neglecting dormancy). Cell‐specific catabolic kinetics are parameterized based on values from microbiological literature (Table S3; Katsev and Halevy 2025). The same set of parameters for each functional microbial group was used for all simulated lakes. Kinetics of the microbially‐catalyzed reactions was thus described without fitting parameters.

**TABLE 1:** Lake‐specific model parameters.

Parameter	Unit	Brownie	Canyon	Cadagno	Malawi	Matano	Comment
T	°C	9	9	6	23	29	At redoxcline
pH	—	7.1	6.9	7.2	7.1	7.2	At redoxcline
${\left[\mathrm{SO}4^{2-}\right]}^{0}$	μM	65	7	350	16	20	Surface sulfate concentration
$F_{\text{FeIII}}$	mmol m^−2^ y^−1^	1500	1500	20	10	300	Flux of Fe(III) to lake surface
$F_{\mathrm{Fe}2}^{L}$	mmol m^−2^ y^−1^	8500	1500	0	0	0	Flux of Fe^2+^ from groundwater
PP	mmol m^−2^ d^−1^	600	60	200	150	40	Integrated gross primary production
$K_{z}^{0}$	m^2^/s	10^−4^	10^−4^	10^−4^	10^−3^	10^−4^	Eddy diffusion coefficient near surface
$H_{K}$	m	1.0	4.0	5.0	40	94	Thermocline depth
$h_{K}$	m	1.2	2.25	1.2	30	6	Thermocline half‐width
$f_{\mathrm{Kz}}$	—	0.01	0.001	0.01	0.01	0.016	$K_{z}$ at thermocline relative to surface
α	m	4	10	3	12	1	Depth scale parameter for $K_{z}$
β	—	6	4	3	2	5.5	Increase in $K_{z}$ in deep monimolimnion
$f_{\mathrm{rec}}$	—	0.9	0.8	0.1	0.05	0.9	Fraction of Fe(III) recycled in sediments
$f_{\mathrm{rec},\mathrm{CH}4}$	—	0.15	0.7	0.1	0.05	0.45	Fraction of POC recycled to CH_4_ in sediments

Vertical transport by water movements in the water column was described using the depth‐dependent turbulent eddy diffusivity, $K_{z}\left(z\right)$. As turbulent mixing is typically high in the lakes' epilimnia, minimal at the thermocline or pycnocline, and sometimes increases slightly with depth due to turbulent energy dissipation near the bottom, the $K_{z}\left(z\right)$ variation with depth z was approximated using the following expression:

(1) $$K_{z}\left(z\right)=K_{z}^{0}\left(f_{\mathrm{Kz}}+\frac{1-f_{\mathrm{Kz}}}{1+e^{\left(z-H_{K}\right)/h_{K}}}\right)+0.1{\left(\frac{z}{\alpha }\right)}^{\beta }\;.$$

Here, z is depth in the water column, $K_{z}^{0}$ is the vertical mixing near the lake surface, the dimensionless factor $f_{\mathrm{Kz}}$ describes the decrease in $K_{z}$ at the thermocline, $H_{K}$ and $h_{K}$ are the depth and width of the thermocline (or pycnocline), and α and β are adjustable parameters that determine the increase in vertical mixing in deeper monimolimnion. We define z and $H_{K}$ from observational studies (see Section 1.1), but other mixing parameters in Equation (1) are poorly constrained. We treat these as model fitting parameters, particularly $f_{\mathrm{Kz}}$, $h_{K}$, α, and β. For Brownie Lake, we match the $K_{z}$ profile to the order of magnitude determined in Lambrecht et al. (2020). Similarly, the $K_{z}$ values in Lake Matano were selected after the modeled results in Katsev et al. (2010). To the authors' knowledge, the remaining study lakes lack quantitative diffusivity estimates at the time of publication. The turbulent eddy diffusion transport was applied to both dissolved and particulate species. Solid particles, in addition, were considered to be transported by the downward Stokes settling ($v_{\text{settle}}$).

Vertical distributions of chemical species, biomasses of functionally defined microbial groups, and the associated rates of biogeochemical reactions were obtained as model solutions by running the model for a sufficiently long time to achieve steady state, usually tens to hundreds of years. In situations where lakes were known to exhibit seasonal variations in their mixolimnia, the model was calibrated by approximating the assumed representative, seasonally averaged distributions. As the sensitivity of model solutions to variations in parameter values is often important for proper interpretations of the results, where possible we comment on the types of obtained solutions, particularly when they exhibit such sensitivity. For a more didactic approach to the sensitivity of the model and its biogeochemical component, the reader is referred to Katsev and Halevy (2025).

### 2.2 Microbiological Analyses

The abundance of methanotrophs and methanogens was determined in Brownie Lake and Canyon Lake using 16S rRNA amplicons generated in Lambrecht et al. (2020). Using methods outlined in Lambrecht et al. (2020), operational taxonomic units (OTUs, 0.03% similarity cutoff) were created with Mothur (Schloss et al. 2009) using the provided Illumina SOP (Kozich et al. 2013) and taxonomically classified to the Silva database v.138 (Quast et al. 2013). Searches for known methanotrophs and methanogens in the taxonomic profiles provided a list of OTUs. The relative abundance of OTUs was calculated in R (Hornik and the R Core Team 2023) using R Studio (RStudio Team 2020) with the decostand function in the vegan library (Okansen et al. 2011). Data processing and graphs were made with the tidyverse library (Wickham et al. 2019). For Brownie Lake, shotgun metagenome libraries were generated using DNA extracted from the chemocline (4–6 m). DNA was extracted using methods outlined by Lambrecht et al. (2020) and sequenced at the University of Minnesota Genomics Core facility using a single lane of Illumina HiSeq 2500 with 2 × 150 base lengths. Individual shotgun metagenome reads were screened for quality and adapters with FastP (Chen et al. 2018). Using the Kbase platform (Arkin et al. 2018), cleaned reads were co‐assembled with MegaHit (Li et al. 2015) and metagenome assembled genomes (MAGs) were created with MetaBat2 (Kang et al. 2019), Concoct (Alneberg et al. 2014), and CocaCola (Lu et al. 2017). Outputted MAGs from the three binning tools were compared and dereplicated with DAStool (Sieber et al. 2018). Dereplicated MAGs were assessed for quality with CheckM (Parks et al. 2014) and taxonomically assigned with GTDB‐tk (Chaumeil et al. 2019) with GTDB database v202. MAGs and the bulk assembly were functionally characterized with DRAM (Shaffer et al. 2020). DRAM profiles were used to search for MAGs capable of sulfur reduction, methane oxidation, or methanogenesis. We recovered no archaeal MAGs, and the mcrA gene, the primary methanogenesis gene, was not found in the bulk assembly. FeGenie was used to identify potential iron‐reducing MAGs (Garber et al. 2020). The abundance of the MAGs was calculated with CoverM (Woodcroft 2007). For downstream metagenome analyses, only MAGs with > 60% completeness and contamination < 10% containing key metabolic indicator genes (e.g., reductive *dsr*AB for sulfate reduction or *mtr*CAB for iron reduction) were used. All raw DNA sequences are available at the National Center for Biotechnology and Information (NCBI) under project number PRJNA560450.

## 3 Results

**FIGURE 2:**
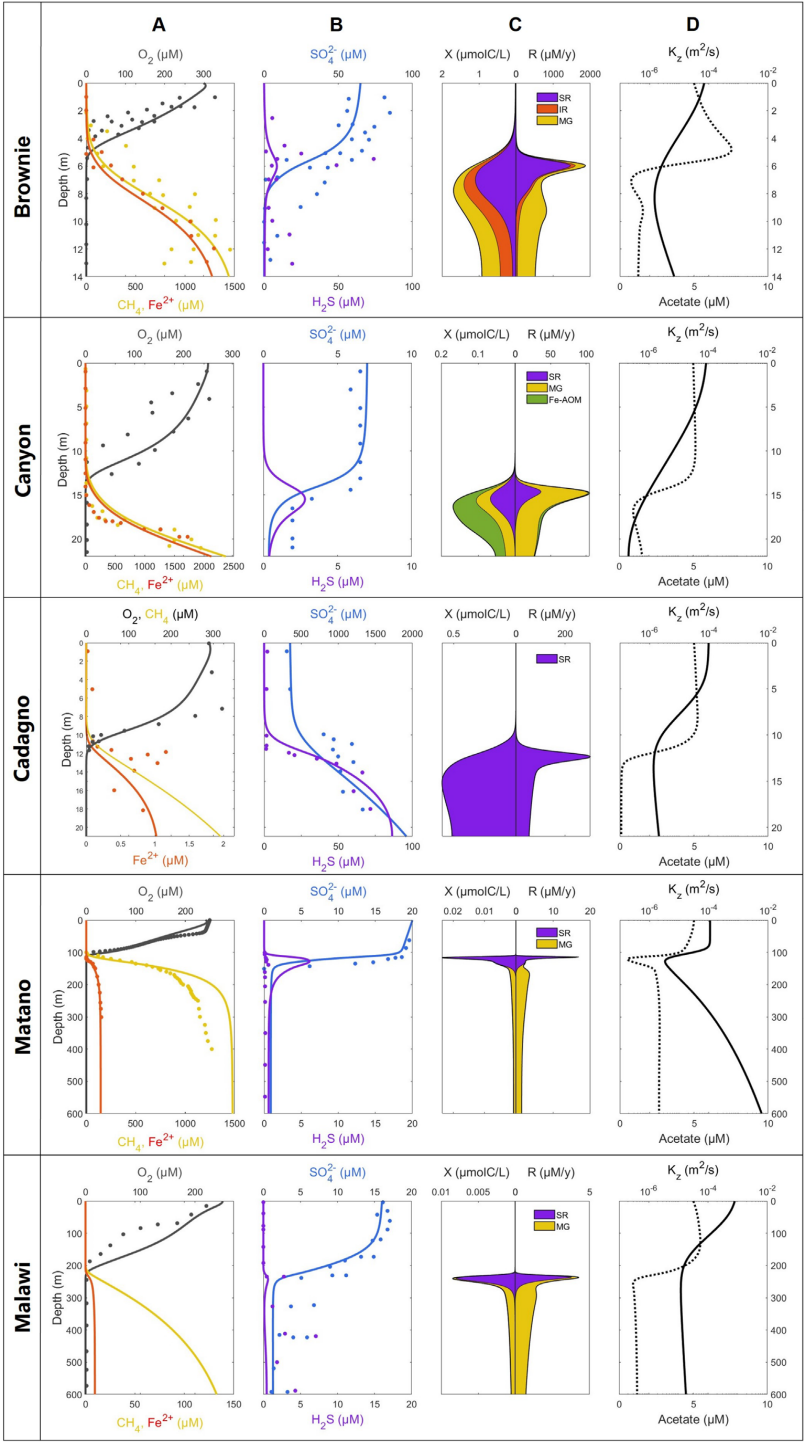
(A, B) Simulated (lines) and observed (datapoints) chemical distributions in study lakes. (C) Simulated microbial biomasses (X) and bulk biogeochemical reaction rates (R) for anaerobic metabolisms. (D) The corresponding depth distribution of the eddy diffusion mixing intensity, $K_{z}\left(z\right)$ (solid lines), and acetate concentrations (dotted lines) in the model. Datapoints are from Lambrecht et al. (2020); Wittkop et al. (2020) (Brownie), Lambrecht et al. (2020) (Canyon), Saini et al. (2022); Xiong et al. (2019) (Cadagno), Crowe, O'Neill, et al. (2008), Crowe, Jones, et al. (2008) (Matano), Li et al. (2018) (Malawi).

Figure 2 shows the simulated distributions of chemical species, microbial biomasses, and metabolism rates for all study lakes, plotted together with observations sourced from literature. We assessed model success by visually fitting profiles to observations. The model successfully reproduced observations in multiple small and medium‐sized lakes (Brownie, Canyon, Cadagno, and Matano) that have relatively constant vertical stratifications amenable to the one‐dimensional approach.

**FIGURE 3:**
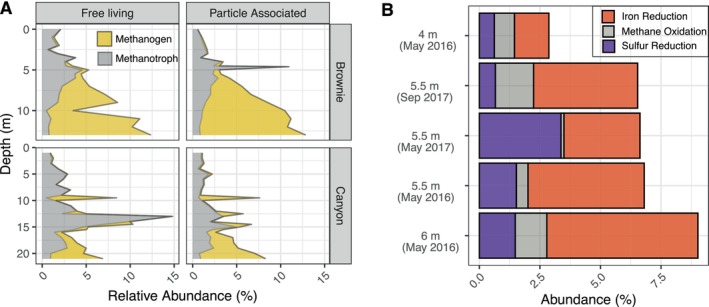
(A) 16S rRNA gene abundance of methanotrophs and methanogens in Brownie Lake and Canyon Lake. (B) Abundance of methanotrophic, sulfur‐reducing, and iron‐reducing Metagenome Assembled Genomes (MAGs) identified in the Brownie Lake chemocline.

In Brownie Lake, the relatively high (60–80 μM) concentrations of sulfate in surface water generate correspondingly high concentrations of hydrogen sulfide in the chemocline. Dissolved sulfide is scavenged in deeper water by very high concentrations of dissolved iron. To account for the observed concentrations of ${\mathrm{Fe}}^{2+}$ in the monimolimnion, the model had to assume the flux of ${\mathrm{Fe}}^{2+}$ through its lower boundary at 8500 mmol/m^2^/y, accounting for a substantial contribution from the sublacustrine groundwater discharge in Brownie Lake (Goudreault 1985; Lambrecht et al. 2018; Akam et al. 2024). Oxidation of ferrous iron at the chemocline, by producing highly reactive Fe(III) oxyhydroxides, supports a substantial population of iron‐reducing bacteria (Figures 2 and 3). The contribution of iron reduction to carbon mineralization, however, is disproportionally small compared to sulfate reduction and methanogenesis. Sulfate reduction in the chemocline and methanogenesis in deeper monimolimnion are predicted to be viable in the water column of Brownie Lake, with correspondingly large microbial populations (Figures 2 and 3). The presence of sulfate reducers and methanogens in our simulations was robust, whereas microbial iron reduction was found to be sensitive to in‐lake conditions (Figure S1). In particular, small adjustments to the thermocline half‐width parameter in Equation (1) produced significant changes to the dominant microbial distributions without significantly affecting the chemical concentration profiles (Figure S1). In contrast to microbially‐catalyzed iron reduction, abiotic iron reduction coupled to the oxidation of hydrogen sulfide produced via sulfate reduction remained active.

Microbial distributions determined in Brownie Lake by 16S rRNA gene abundance (Figure 3A.) broadly match the simulated patterns. Both methanotrophs (methane consuming) and methanogens (methane generating) OTUs were found at similar abundances in the free‐living (0.22–3.0 μm filter pore size) and particle associated (> 3.0 μm filter pore size) fractions. Methanogens in both filter fractions were found at similar relative abundances, increasing with depth in the monimolimnion and exceeding the abundances of sulfate reducers below the chemocline, also consistent with the simulation results in Figure 2. Metagenome Assembled Genomes (MAGs) capable of Fe reduction, methane oxidation, and sulfur reduction were identified within the chemocline (4–6 m, Figure 3B.), and were also quantitatively consistent with model predictions. Measured abundances of all anaerobic taxa were higher in September, when the oxycline is located higher in the water column, than in May.

In Canyon Lake, lower concentrations (7 μM) of surface sulfate generate correspondingly lower concentrations of sulfide in the deep chemocline, lower rates of sulfate reduction, and lower biomasses of sulfate reducing bacteria. As in Brownie Lake, sublacustrine inputs of iron were required in the model to reproduce the high (mM level) observed concentrations of ${\mathrm{Fe}}^{2+}$ in bottom waters, as internal recycling of iron in sediments alone was insufficient to achieve such high concentrations. Simulations predicted a possible presence of Fe‐dependent AOM microbial consortia in Canyon Lake, which was the only such system among our study lakes. The putative presence of Fe‐AOM, however, was highly sensitive to mixing rates, and the pathway could disappear from the model solutions when mixing rates were varied even slightly (Figure S1). Measured abundances of methanogens (Figure 3A) increased immediately below the oxycline, broadly in agreement with simulations. Abundances and distributions of methanogens varied between the free‐living and particle‐associated fractions and with depth. Shotgun metagenomes were not generated for Canyon Lake, and unfortunately, 16S rRNA analysis does not provide gene evidence for Fe‐AOM. However, Fe‐AOM has been observed in anoxic freshwater systems where bacterial methanotrophs are highly abundant and iron reducing conditions are present (Li et al. 2023; Zheng et al. 2020). This suggests that Fe‐AOM could be present in Canyon Lake but needs further investigation.

Sulfate reduction predictably dominated in Lake Cadagno where sulfate concentrations within the redoxcline exceed 400 μM (Figure 2). Hydrogen sulfide concentrations around 70 μM were reproduced by the model, while ${\mathrm{Fe}}^{2+}$ concentrations remained suppresed to sub‐micromolar levels in agreement with observations. To reproduce the mM levels of sulfate in bottom waters, the model required imposing an external flux of ${{\mathrm{SO}}_{4}}^{2-}$ into the deep waters at 7000 mmol/m^2^/y.

Lake Matano simulations with the relatively low surface sulfate (18–20 μM) show that sulfate reduction generates a 5 μM hydrogen sulfide peak and supports the sulfate‐reducing microbial populations in the chemocline. Despite the ferruginous deep monimolimnion, methanogenesis is indicated to be the dominant metabolic pathway there. This is similar to the viability of methanogenesis in ${\mathrm{Fe}}^{2+}$ rich Brownie Lake and Canyon Lake. Below 150 m, the model overestimates ${\mathrm{CH}}_{4}$ concentrations by 200–300 μM Crowe, O'Neill, et al. (2008). This was the consequence of using the same model and the parameter set (Table S3) as for all other lakes. The analogous simulation in Katsev and Halevy (2025) by a version of the model that was slightly more tailored to the conditions specifically in this lake produced a better result.

In the large Lake Malawi, the model reproduced the distributions of oxygen and sulfate and the mid‐water layer of low‐level euxinia. It could not reproduce the absence of ${\mathrm{Fe}}^{2+}$ in the anoxic deep waters. Although in 2013 ${\mathrm{Fe}}^{2+}$ was not detected in deep monimolimnion and concentrations at the sediment–water interface in deep sediments were as low as 1–2 μM (Li et al. 2018), our steady state model consistently generated accumulation of ${\mathrm{Fe}}^{2+}$ to around 10 μM, if the observed sulfide distribution was to be reproduced. Adjusting model parameters to lower the ${\mathrm{Fe}}^{2+}$ concentrations in the deep water caused the mid‐water euxinic zone to grow both in vertical extent and concentration values, departing from observations. The greater‐than‐observed concentration of bottom‐water iron obtained in a model that was propagated to steady state is not necessarily contradictory and could indicate that the conditions in the lake are currently transitory. Accumulations of dissolved iron at similar concentrations in a smaller Lake Towuti, for example, were suggested to take place over about a decade (Pu et al. 2025). Given the large volume of Lake Malawi's monimolimnion, accumulation of iron even to these low concentrations (e.g., after an episodic weak ventilation) would require hundreds to thousands of years. Alternatively, the effects of three‐dimensional basin‐scale mixing in Lake Malawi (Katsev et al. 2017) may not be adequately accounted for by our one‐dimensional model. An additional factor to consider is the potential effect of the nitrogen cycle, which is not considered by the present model (see Supporting Information). Nitrate in Lake Malawi only reaches 5 μM and is exhausted by 250–300 m depth (Li et al. 2018). As the measured concentration profiles indicate the zone of sulfate reduction to be around 250–500 m (Li et al. 2018), we do not expect this factor to have a significant influence on the bulk water chemistry in the monimolimnion. It could, however, shift the simulated sulfate profile deeper (Figure 2), for a better agreement with observations, and could affect the abundances of sulfate reducers in the region of overlap with nitrate.

Similarly to our results in Lake Matano, the model for Lake Malawi predicted sulfate reduction and methanogenesis to dominate the organic carbon mineralization (Figure 2). This is despite significant quantities of reducible iron in Lake Malawi sediments (Li et al. 2018) which must survive reduction during its transport to the bottom.

## 4 Discussion

### 4.1 Partitioning of Anaerobic Metabolisms

The model's ability to reproduce essential features of chemical stratifications using the same set of microbial kinetics parameters for multiple lakes suggests a broad applicability of the approach. In contrast to traditional reaction‐transport models that tend to be overparameterized with respect to reaction kinetics, the kinetics of microbially‐catalyzed reactions are parameterized here based on cell‐level microbiological data, with no fitting parameters. Fitting the concentration profiles in Figure 2 involved setting lake‐specific boundary conditions and finding a suitable approximation for physical mixing, $K_{z}\left(z\right)$. The biomass‐explicit approach used here for describing microbial kinetics and growth (see Supporting Information) largely follows one of several previously suggested frameworks (Jin 2023) and relies on several assumptions. In particular, it assumes that biomass synthesis and catabolism are tightly coupled (Equation S3). It also does not explicitly take into account the possible limitation of microbial growth by nutrient (e.g., P) availability, which is usually justified in nutrient‐rich monimolimnia. The kinetics of all microbially‐catalyzed reactions is described here using Monod‐type expressions (Equation S5), which are generally accepted to work well. Surface‐controlled reactions (e.g., reduction of solid ferric oxides) may be better described mechanistically by the functionally similar Contois equation, which uses the concentration ratio of substrate (S) to biomass (X) (Jin 2023). The Contois equation effectively makes the Monod half‐saturation constants for solid substrates, such as $K_{\text{FeIII}}$ (Table S3), biomass‐dependent. It is not clear, however, if these finer distinctions are significant enough to cause practical difficulties in describing biogeochemical rates in water columns.

Fitting the vertical distributions of chemical species in a water column does not necessarily imply fully constraining reaction kinetics. Microbially catalyzed biogeochemical reactions occur at their highest rates in narrow chemoclines, whereas rates in voluminous but more chemically homogeneous deeper strata can be low. The profiles of chemical species across monimolimnia may instead be strongly shaped by depth‐varying rates of physical mixing, with sources and sinks of chemicals being restricted mainly to sediments and the chemocline (Crowe, O'Neill, et al. 2008). In such cases, observed chemical distributions provide only a weak constraint on chemical rates and microbial activities (Louca et al. 2019).

Nevertheless, biomass‐explicit modeling offers verifiable predictions about the relative abundances of metabolically defined microbial groups. The previous application of the method by Katsev and Halevy (2025) involved only limited verification against available data and could not verify predictions for the distributions of microbial abundances. Our present results support the validity of the method, which appears to provide adequate descriptions for the proportions of iron reducers, sulfate reducers, and methanogens in Brownie Lake (Figure 3). We want to point out that microbial abundances from metagenomes do not directly translate to biomass estimates, process rates, or activity. The model‐predicted rates of methanogenesis, inferred to be disproportionately large compared to the share of the methanogenic archaeal biomass, are also in line with recent independent estimates in Brownie Lake (Akam et al. 2024). Gene abundances and expression rates, in particular, can be used to validate the model predictions (e.g., Louca et al. 2016).

Our simulations, combined with previous theoretical insights (Katsev and Halevy 2025), make several important predictions. They indicate, in particular, that sulfate reduction can dominate anaerobic mineralization of organic carbon even in ferruginous environments rich in reduceable iron (Figure 2). At tens of micromolar sulfate, the produced hydrogen sulfide does not accumulate, as it is efficiently scavenged within the chemocline by solid Fe(III) via abiotic oxidation to $S^{0}$, and in deeper ferruginous waters by ${\mathrm{Fe}}^{2+}$ via precipitation of solid sulfides. A significant proportion of dissolved ferrous iron in ferruginous lakes likely comes from sediments and/or groundwater, with minimal contribution from microbial iron reduction in the water column. Settling of Fe(III) particles through the anoxic water column is fast enough to prevent their reductive dissolution, and sediments of non‐euxinic lakes are recognized to have high concentrations of reduceable iron (Li et al. 2018). In our modeled lakes, concentrations of dissolved iron exceeding several tens of micromolar (e.g., in Brownie Lake and Canyon Lake) could only be achieved in presence of sub‐lacustrine (groundwater) external sources of iron, which are hypothesized to exist within those lakes (Kniptash et al. 2025).

Methanogenesis is similarly viable in ferruginous water columns (Katsev and Halevy 2025), and is expected to occur primarily below the zone of active sulfate reduction. Methanogenic archaea are known to have higher cell‐specific rates of catabolic carbon utilization than iron reducers (Scholten et al. 2002; Bonneville et al. 2004), which may well offset their energetic disadvantage of generating less catabolic energy per carbon atom. Our observations of active (Lambrecht et al. 2020) and dynamic methanogenic populations in Brownie Lake and Canyon Lake (Figure 3) support this conclusion, and align well with previous inferences of active water‐column methanogenesis in Brownie Lake (Akam et al. 2024). Substantial fluxes of methane must be coming from sediments as well, produced by the mineralization of organic matter to ${\mathrm{CO}}_{2}$, $H_{2}$, acetate, or other alcohol and subsequent conversion to methane. In contrast to ${\mathrm{Fe}}^{2+}$, the observed high (mM level) concentrations of methane in the monimolimnia of lakes Matano, Brownie, and Canyon could be reproduced by considering the in‐lake sedimentary recycling of organic matter alone ($f_{\mathrm{rec},\mathrm{CH}4}$ in Table 1), without the need for external sources of methane. It is not known whether the same groundwater sources that supply iron to those lakes also supply methane. Methanogenic production in organic‐rich sediments, however, could plausibly dominate over the rates of supply from the organic‐poor catchment rocks. In shallow lakes such as Brownie and Canyon, additional methane is known to be released via ebullition (Lambrecht et al. 2020).

### 4.2 Are Medium‐Sulfate Systems Rare?

A survey of meromictic lakes across available literature (e.g., Swanner et al. 2020; Schultze et al. 2017) and online sources revealed an intriguing scarcity of meromictic lakes with medium‐range surface sulfate concentrations of several hundred μM. Lakes that do fall into this range, such as Lake Cadagno (Figure 2), tend to have atypical vertical structures: sulfate supplied into the deep waters by groundwater reaches high concentrations near the bottom, and the surface serves as a sink rather than a source for sulfate (Yoshimura et al. 2020). This apparent gap in natural lacustrine sulfate concentrations is puzzling. Similarly to non‐meromictic lakes (Zak et al. 2021), one might expect to find a continuum of surface sulfate concentrations corresponding to continua of both local catchment geologies and bottom water sulfide concentrations. In a simple paradigm where production of hydrogen sulfide by sulfate reduction overpowers the combined external and internal sources of ${\mathrm{Fe}}^{2+}$ at around 100 μM of sulfate at lake surface, lakes with higher sulfate levels would develop euxinic bottom waters. The surface sulfate levels, however, would be unaffected by the redox state of the bottom waters in this scenario.

**FIGURE 4:**
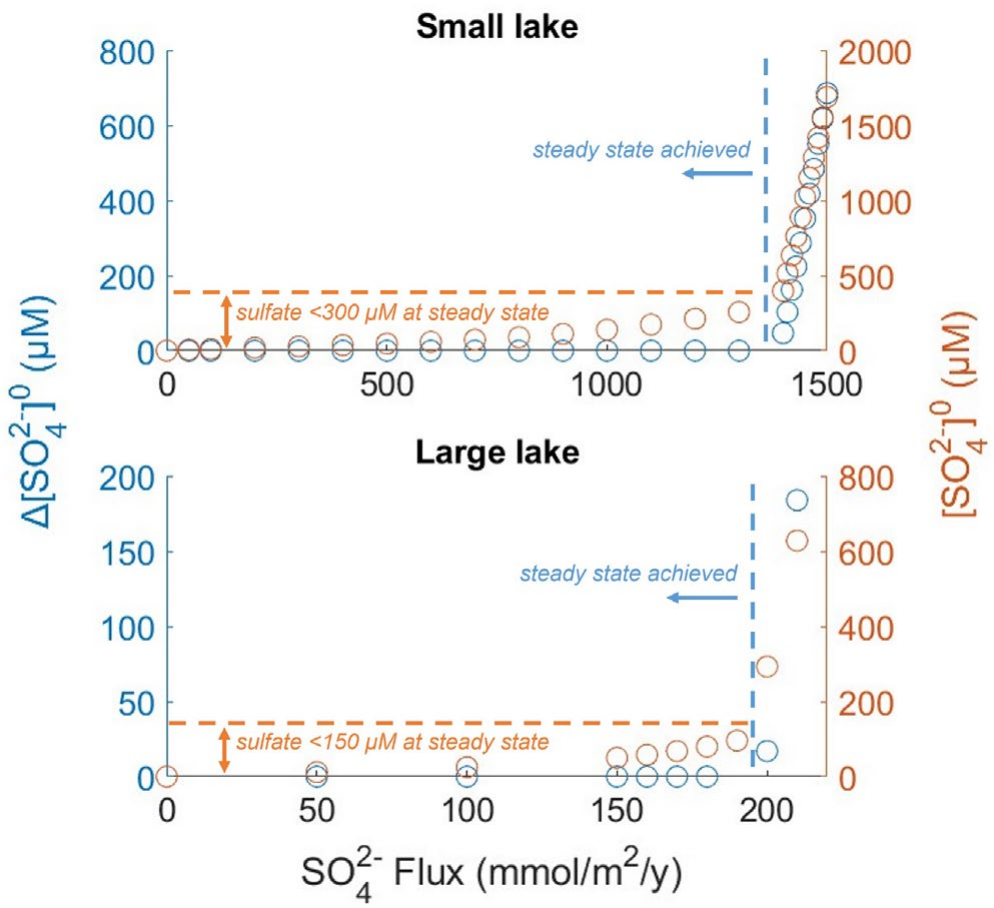
Above several hundred μM, sulfate accumulates to mM levels without reaching steady state. Sulfate accumulates when its inputs into the lake exceed the rates of S removal by processes such as burial of sulfide minerals into sediments. $\Delta {\left[{{\mathrm{SO}}_{4}}^{2-}\right]}^{0}$ is the change in surface sulfate concentration between subsequent model runs; zero values indicate steady state. ${\left[{{\mathrm{SO}}_{4}}^{2-}\right]}^{0}$ are the corresponding sulfate concentrations at lake surface at the simulation end.

An investigation using our model offers a non‐trivial clue to this apparent gap in the continuum of lacustrine sulfate concentrations (Figure 4). The model reproduces this gap when the boundary condition for sulfate at the lake surface is changed from imposed‐concentration to imposed‐flux, which better represents the effect of S inputs from the watershed. Such boundary condition allows for a dynamic accumulation of sulfate. When sulfate is abundant to produce a sufficient quantity of hydrogen sulfide to remove ${\mathrm{Fe}}^{2+}$ from bottom waters, further removal of H2S into sediments becomes impossible. The reoxidation of sulfide and the repeated cycling of sulfate and sulfide across the redoxcline then leads to the accumulation of S in the lake. Surface sulfate concentrations continue to rise, until sulfate inputs from the watershed can be balanced by the next strongest sink, such as removal with surface outflow from the lake, degassing of H2S into the atmosphere, or burial of mineral S phases such as gypsum, which forms under much higher (mM) sulfate levels. (Surface outflow and gypsum precipitation were not considered by the present model.) The magnitude of the external surface sulfate flux and the time scale of this runaway transition to euxinia depend on the particulars of the given aquatic system, such as its size and rates of sulfur transport within its water column. Figure 4 illustrates this effect simulated for a “small lake” (using the Brownie Lake model without the groundwater inputs of iron) and a “large lake” (using the Lake Malawi model). The figure shows changes in the surface sulfate concentrations ($\Delta {\left[\mathrm{SO}4^{2-}\right]}^{0}$) between two consecutive runs of the model with the same sets of parameters, each run lasting 100 simulation years for the “small lake” and 10,000 years for the “large lake”. The $\Delta {\left[\mathrm{SO}4^{2-}\right]}^{0}$ value of zero indicates that a steady state was achieved in this time frame, whereas a positive value indicates continuous accumulation of sulfate. Whereas the critical sulfate flux leading to runaway behavior depended on the system, sulfate concentrations at the lake surface remained below the “medium range” of several hundred μM whenever a steady state could be achieved, and evolved to very high (mM) levels when it could not (Figure 4).

### 4.3 Importance of Physical Mixing for Microbial Conditions

Our simulations illustrate the importance of hydrodynamic conditions, such as the rates of physical mixing, for the development of chemical and microbiological structures within stratified water columns. Microbial population abundances are commonly linked to chemical conditions, as substrate availability, pH, and Eh all affect the growth potential of microbial cells. Physical processes, however, control the supply of chemicals into microbial reaction zones. They also affect the length of time that substrates and microbial cells remain in the reaction zone where conditions are favorable for microbial growth. In some lakes, relatively small changes in physical mixing may amount to large qualitative changes in microbial activity, as outcomes of microbial competitions can have sharp thresholds (Katsev and Halevy 2025). Figure S1 illustrates how small changes in $K_{z}\left(z\right)$ can bring about large changes in microbial rates and biomasses in the water column. In Canyon Lake, methanogens dominate for a thermocline with the half‐width of $h_{K}=2.3$ m, whereas Fe‐AOM is the preferred pathway for $h_{K}=2.13$ m. In Brownie Lake, methane reduction dominates at both $h_{K}=1.2$ m and $h_{K}=1.32$ m, but iron reduction is no longer significant for $h_{K}=1.32$ m.

### 4.4 Development of Ferruginous vs. Euxinic Conditions

Our results support the idea that the development of ferruginous or euxinic conditions in a stratified anoxic water column depends on the mass balance between the inputs of S and highly reduceable iron (${\mathrm{Fe}}_{\mathrm{HR}}$). Poulton and Canfield (2011) proposed that Phanerozoic oceans may have been precariously balanced between ferruginous and euxinic conditions (when anoxic), as sulfur and iron were supplied to the ocean at a near critical $S:{\mathrm{Fe}}_{\mathrm{HR}}$ ratio of about 1.8, which corresponds to the typical elemental ratio in sulfides deposited under euxinic conditions. The implication is that minor variations in sulfur or iron inputs could shift the chemical balance of the ocean towards either a ferruginous or a euxinic state. Variations in the respective burial rates, for example, in response to the extent of ocean anoxia, could create further mechanisms for shifting the balance and mass balance‐induced tipping points (Fakhraee et al. 2024).

The mechanism implicated here does not involve the bistability suggested by the box‐type and zero‐dimensional models of van de Velde et al. (2020). Instead, in our spatially‐explicit model, transitions from a Fe‐dominated water column to a sulfide‐dominated one occur by a gradual growth of the mid‐water euxinic zone and the corresponding gradual titration of ${\mathrm{Fe}}^{2+}$ from the bottom water. Increasing the inputs of sulfate relative to those of reactive iron, nevertheless, does cause a tipping‐point transition in the concentration of sulfate that accumulates in the surface waters over time. This stabilizes the already predominantly euxinic environments and makes them unlikely to persist at medium‐range sulfate. At lower sulfate concentrations, the abiotic reduction of iron oxides by hydrogen sulfide may create conditions for a hysteresis whereby dynamic transitions from a more sulfide‐rich state to a more iron‐rich one are more difficult than in the opposite direction. For the long‐term balance between the water‐column content of dissolved iron vs. dissolved hydrogen sulfide, however, the mass balance between the inputs of S and Fe into the water body provides a more straightforward and robust effect.

Our results in modern meromictic lakes offer a few additional important insights. The threshold ratios for the S and Fe inputs into the water body at which regime shifts occur depend on the available sinks for either element. For instance, degassing of hydrogen sulfide into the atmosphere (Crémière et al. 2024) may be non‐negligible, even in the well‐mixed epilimnion where the sulfide concentrations are low. In lakes, substances can also be removed by river outflows on time scales comparable to the hydrological residence time. Furthermore, for ferruginous conditions to develop, the total iron inputs into the water body need to outpace not the inputs of sulfate per se, but rather the capacity of the system for sulfate reduction that produces dissolved hydrogen sulfide. This makes the thresholds in the S:Fe input ratios system‐specific. Hydrogen sulfide binds ${\mathrm{Fe}}^{2+}$ in solid sulfides and reacts abiotically with Fe(III) particles in the water column, preventing their microbial reduction. The production of hydrogen sulfide by sulfate reduction, importantly, depends on the transport of sulfate from surface waters into the anoxic zone, across the typically quiescent chemocline. The capacity of the water body for sulfide production thus depends on the hydrodynamics of the environment. Above a certain lake‐specific $S:{\mathrm{Fe}}_{\mathrm{HR}}$ input threshold, sulfate can begin to accumulate in the surface mixed layer. With the exception of very oligotrophic lakes where sulfate reduction may be limited by availability of organic matter, this further enhances the production of hydrogen sulfide below the redoxcline, leading to full water column euxinia.

### 4.5 Implications for Ancient Oceans and Their Modern Analogues

Estimates for chemical concentrations in early oceans have been broadly informed by processes in analogous modern systems (Crowe et al. 2014). If conditions in modern lakes were to be taken phenomenologically as quantitative predictors for coastal water columns in early oceans, ferruginous conditions could be considered likely to persist in bottom waters when sulfate concentrations in the surface mixed layer were below 100 μM. At higher sulfate levels, euxinic conditions would extend from the oxycline to the bottom. Similar concentration ranges for surface sulfate (< 400 μM) were obtained in simplified ocean models that considered the conceptually similar vertical cycling of sulfur (Fakhraee et al. 2019). It is a pertinent question, though, whether the mechanism identified here that makes it difficult for sulfate to stabilize at several hundreds of μM in lakes would apply analogously to early oceans. If sulfur inputs to the ocean exceeded the finite burial fluxes of pyrite over sufficiently long time scales, it seems reasonable that sulfate concentrations would increase until they were balanced by the next strongest sink (e.g., precipitation of barite or gypsum). This implies that, in a non‐ferruginous ocean, sulfate concentrations are likely to rise into the mM range, rather than remain at several hundreds of μM. The dynamic mechanism of sulfur recycling that is responsible for sulfate accumulation was not considered by previous Proterozoic ocean models (Fakhraee et al. 2019). The runaway accumulation of sulfide observed in our model at higher inputs of sulfate would then suggest that the redox state of ancient oceans may have been bimodal: either ferruginous with surface sulfate concentrations not exceeding tens of micromolar, or sulfidic with sulfate levels rising to mM concentrations over geologically short times and sulfide accumulating in wide strata of the ocean. Depending on the strengths of hydrothermal inputs of Fe into the deep ocean, deep bottom waters may have maintained ferruginous conditions. The time scales on which a sulfate‐rich ocean can switch to a ferruginous low‐sulfate state have been shown to be geologically short, on the order of a few million years (Bauer et al. 2022).

Under ferruginous conditions, our simulations suggest that dissolved iron concentrations in deep waters are unlikely to exceed hundreds of μM, unless supported by strong external fluxes of iron, such as from groundwater. In the case of coastal regions in early oceans, this iron flux could originate from the pelagic deep ocean reservoir (Katsev and Halevy 2025) or hydrothermal inputs (Poulton and Canfield 2011). Despite ferruginous conditions, our simulations suggest that sulfate reducers and methanogens could have dominated in early oceans (Figure 2), at least during geologic periods when the surface mixed layer contained oxygen and could not support appreciable photoferrotrophy (Katsev and Halevy 2025).

## 5 Conclusions

The modeling approach that considers the energetics, kinetics, and competition among microbial populations (Katsev and Halevy 2025) appears to be successful in reproducing key biogeochemical features in multiple meromictic lakes that vary in size, geographic location, and water chemistry. The model employed a constant set of pathway‐specific microbial parameters, with physical mixing being an important adjustable parameter. The simulated microbial biomasses and reaction rates for the anaerobic pathways of iron reduction, sulfate reduction, and methanogenesis align favorably with the available observational data. The results suggest that: (1) In deep anoxic monimolimnia, sulfate reduction and methanogenesis can dominate carbon mineralization even under high iron content; (2) In dynamic water columns that experience seasonal variations, cell abundances for dominant microbial pathways may fluctuate sensitively, so their predictions may be possible only in a course‐grained sense (Moran and Tikhonov 2022). Predicted patterns of microbial activity may hold statistically across similar types of environments but may not necessarily agree quantitatively with any single dataset taken at a given moment in time; (3) The vertical extents and intensities of euxinic and ferruginous strata are regulated by the balance between the availability of reactive iron oxides and the system's capacity for sulfate reduction. Besides the availabilities of sulfate and organic matter, the latter factor depends on the rates of physical transport across the chemocline and on other potential sinks for sulfate, making the S:Fe input ratios at which the regime transitions occur system‐specific; (4) The finite nature of the iron sulfide sink creates a tipping point whereby sulfate accumulates to mM concentrations once its inputs into the water body exceed the rates of S removal by burial into sediments. This may explain the apparent scarcity of meromictic lakes with medium‐sulfate levels (100 s of μM). Predominance of ferruginous vs. euxinic conditions in the stratified anoxic oceans of the Archean and Proterozoic eons may be constrained by a similar mechanism.

## Conflicts of Interest

The authors declare no conflicts of interest.

## Supporting information


**Data S1:** gbi70037‐sup‐0001‐Supinfo.pdf.

## Data Availability

The data that support the findings of this study are available from the corresponding author upon reasonable request.
