# Supplementary material for: Understanding Ferruginous Versus Euxinic Conditions by Simulating Microbial Conditions in Meromictic Lakes
Source: Geobiology. 2025 Oct 20;23(5):e70037. doi: 10.1111/gbi.70037 (PMC12536669; doi:10.1111/gbi.70037)
Supplement: Supplementary file 1 — Data S1: gbi70037‐sup‐0001‐Supinfo.pdf. [file GBI-23-e70037-s001.pdf]

Supporting Information

Understanding Ferruginous versus Euxinic Conditions by Simulating Microbial Conditions in Meromictic Lakes

Vanessa M. Hawkins*[,1,4], Cody S. Sheik[2,3] and Sergei Katsev*[,1,3]

[1]Department of Physics and Astronomy, University of Minnesota Duluth, Duluth, Minnesota, USA
[2]Department of Biology, University of Minnesota Duluth, Duluth, Minnesota, USA
[3]Large Lakes Observatory, University of Minnesota Duluth, Duluth, Minnesota, USA
[4]Present address: College of Earth, Ocean, and Atmospheric Sciences, Oregon State University, Corvallis, Oregon, USA

*Corresponding authors: skatsev@d.umn.edu; hawkinsv@oregonstate.edu

Biogeochemical reaction-transport model

This study uses the model of Katsev and Halevy (2025), with minor modifications introduced as required for modeling meromictic lakes. For completeness, the description of the model is reproduced below. Differences from the model of Katsev and Halevy (2025) are stated in Table 1 in the main manuscript and explicitly in the text below, where needed.

Bioenergetics and microbial kinetics The model follows the conceptual approach of González-Cabaleiro *et al.* (2016) and Smeaton and Van Cappellen (2018). The net change in the Gibbs free energy of a microbial metabolism (per C-mol of new biomass) is

$$\Delta G = \lambda_{cat}\Delta G_{cat} + \Delta G_{ana} \tag{1}$$

where ΔG_{cat} and ΔG_{ana} are, respectively, the Gibbs free energy of the catabolism and anabolism, and λ_{cat} is the number of times a catabolic reaction needs to be run to provide the energy needed to form one C-mol of biomass. The maximum growth yield (mol biomass synthesized per mol reaction) is then

$$Y_{max} = \frac{1}{\lambda_{cat}} = \frac{\Delta G_{cat}}{\Delta G - \Delta G_{ana}} \tag{2}$$

We follow the argument of Heijnen and Van Dijken (1992) that the Gibbs free energy of a macrochemical reaction ΔG is equivalent in magnitude to the dissipated energy ΔG_{dis}. The ΔG_{dis} was calculated using the phenomenological expression of Heijnen and Van Dijken (1992) for heterotrophic and autotrophic metabolisms. ΔG_{cat} and ΔG_{ana} were calculated for each individual reaction from the corresponding standard energies of formation and activity products, at relevant environmental conditions.

The cell-specific catabolic rates were calculated as functions of substrate (S) concentrations:

$$r = \frac{1}{X}\frac{dS}{dt} = F_K F_T \tag{3}$$

Here X is the biomass in C-mols. The kinetic factor F_K depends on the pathway-dependent maximum cell-specific rate V_{max} and expresses the Monod-type dependence on substrate concentrations:

$$F_K = V_{max}\prod \frac{S_i}{K_m^i + S_i} \tag{4}$$

To account for oxygen toxicity, F_K expressions for anaerobic pathways also included the inhibition factor $K_{O_2}^{inh}/(K_{O_2}^{inh} + [O_2])$.

The thermodynamic factor F_T is (Jin and Bethke 2007)

$$F_T = 1 - \exp\left(\frac{\Delta G_{cat} + m\Delta G_{ATP}}{\chi RT}\right) \tag{5}$$

where ΔG_{ATP} is the energy being conserved into ATP, and m and χ are the metabolism-specific stoichiometric factors (Table S3). The evolution of the chemical (S) and biomass concentrations (X) are then described as:

$$\frac{dS_s}{dt} = A_{sm}r_{mb}X_b + B_{sn}Y_{nm}r_{mb}X_b \tag{6}$$

$$\frac{dX_b}{dt} = \sum_n Y_{nm}r_{mb}X_b - \lambda(m_G)X_b \tag{7}$$

Here, summation is implied over the repeating indices (the Einstein convention). A_{sm} is the stoichiometric matrix for substrate s in a catabolic reaction m catalyzed by the microbial population b; B_{sn} is the stoichiometric matrix for the use of substrate s in an anabolic growth reaction n, Y_{nm} is the yield for the microbial growth utilizing catabolic reaction m and anabolic reaction n. The natural decay of the biomass is described by the "death" term λ, which is a function of the maintenance energy m_G, which itself depends on the metabolic power (see below). For simplicity, in the present model, each catabolic reaction was considered catalyzed by a separate microbial population, with either heterotrophic or autotrophic anabolism. This approach was used to describe the microbial iron reduction (assuming goethite as the reactive solid phase), sulfate reduction, methanogenesis, and Fe- and S-dependent AOM. Kinetic formulations for other reactions are listed in Table S2.

Reaction-transport model The one-dimensional reaction transport model solved a system of partial differential equations for a set C_j of substrate concentrations and biomasses:

$$\frac{\partial C_j}{\partial t} = \frac{\partial}{\partial z}\left(K_z(z)\frac{\partial C_j}{\partial z}\right) - v_j\frac{\partial C_j}{\partial z} + \sum_i \nu_i R_{ij} \tag{8}$$

Here, $K_z(z)$ is the depth-dependent turbulent eddy diffusion coefficient, v_j is the settling velocity, and R_{ij} are the rates of reactions, with stoichiometric coefficients ν_i. Explicitly considered concentrations included those of POM, Ac, O_2, SO_4^{2-},$\Sigma H_2S = H_2S + HS^-$, FeOOH, Fe^{2+}, S^0, FeS, and the biomasses of the microbial populations that catalyze the corresponding reactions: X_{IR}, X_{SR}, X_{CH4}, X_{S-AOM}, X_{Fe-AOM}. The concentrations of DIC and ammonium (as the nutrient for anabolism), as well as the pH, were prescribed and

Table. S1 Reactions included in the model. The Gibbs free energy is in units of kJ mol^{-1} at pH 7.

Reaction	ΔG^0
$CO_2 + H_2O + light \longrightarrow POM + O_2$	
$POM \longrightarrow CH_3COO^-$	
$CH_3COO^- + 2\,O_2 \longrightarrow 2\,HCO_3^- + H^+$	
$CH_3COO^- + SO_4^{2-} \longrightarrow 2\,HCO_3^- + HS^-$	-48
$CH_3COO^- + 8\,FeOOH + 15\,H^+ \longrightarrow 8\,Fe^{2+} + 2\,HCO_3^- + 12\,H_2O$	-132
$CH_3COO^- + H_2O \longrightarrow HCO_3^- + CH_4$	-15
$SO_4^{2-} + CH_4 \longrightarrow HS^- + HCO_3^- + H_2O$	-33
$8\,FeOOH + CH_4 + 15\,H^+ \longrightarrow 8\,Fe^{2+} + HCO_3^- + 13\,H_2O$	-117
$H_2S + 2\,O_2 \longrightarrow SO_4^{2-} + 2\,H^+$	-830
$4\,Fe^{2+} + O_2 + 6\,H_2O \longrightarrow 4\,FeOOH + 8\,H^+$	-371
$CH_4 + 2\,O_2 \longrightarrow HCO_3^- + H_2O + H^+$	-859
$2\,FeOOH + H_2S + 4\,H^+ \longrightarrow S^0 + 2\,Fe^{2+} + 4\,H_2O$	-32
$Fe^{2+} + HS^- \longrightarrow FeS + H^+$	
$4\,S^0 + 4\,H_2O \longrightarrow SO_4^{2-} + 3\,HS^- + 5\,H^+$	+55
$0.525\,CH_3COO^- + 0.2\,NH_4^+ + 0.275\,H^+ \longrightarrow$	
$\longrightarrow X_{hetero} + 0.05\,HCO_3^- + 0.4\,H_2O$	+612
$CO_2 + 0.2\,NH_4^+ + 0.6\,H_2 \longrightarrow X_{auto} + 0.2\,H^+ + 1.5\,H_2O$	+543

Table. S2 Reaction rate formulations. All reaction rates are in units of μM y^{-1}. R_{PPO2}, R_{PPFe} and R_{PPH2S} are, respectively, the rates of oxygenic, iron-driven anoxygenic, and sulfide-driven anoxygenic photosynthesis. R_PP is the rate of gross primary production. R_G is the rate of particulate organic matter (POM) hydrolysis. R_{O2} is the rate of aerobic respiration. R_{HSox}, R_{Feox}, and R_{CH4ox} are, respectively, the rates of sulfide, ferrous iron, and methane oxidation by oxygen. $R_{HSFeIII}$ is the rate of sulfidization of Fe(III) (oxyhydr)oxides. R_{FeS} is the rate of FeS precipitation, which depends on the degree of FeS saturation, Ω_{FeS}. R_{disp} is the rate of elemental sulfur disproportionation.

Rate
$R_{PPO2} = R_{PP} \cdot f_{oxygenic}$
$R_{PPFe} = R_{PP} \cdot (1 - f_{oxygenic}) \frac{[Fe^{2+}]}{[Fe^{2+}]+K_{PFe}}$
$R_{PPH2S} = R_{PP} \cdot (1 - f_{oxygenic}) \frac{[\Sigma H_2S]}{[\Sigma H_2S]+K_{Ps}}$
$R_G = k(z, [O_2])[POM]$
$R_{O2} = R_G \frac{[O_2]}{[O_2]+K_{O2}}$
$R_{HSox} = k_{HSox}[\Sigma H_2S][O_2]$
$R_{Feox} = k_{Feox}[Fe^{2+}][O_2]$
$R_{CH4ox} = k_{CH4ox}[CH_4][O_2]$
$R_{HSFeIII} = k_{HSFeIII}[FeOOH][\Sigma H_2S]$
$R_{FeS} = k_{FeS}(\Omega_{FeS} - 1)$
$\Omega_{FeS} = [Fe^{2+}][HS^-]/([H^+]K_{FeS})$
$R_{disp} = k_{disp}[S^0]$

assumed constant. The kinetics of the biomass-explicit reactions were simulated as described above for the bioenergetics model.

The boundary conditions were prescribed as fixed-concentrations for the dissolved species and fixed-fluxes for the particulate species. A no-gradient boundary condition was imposed at the bottom of the domain, except for species such as Fe^{2+} and CH_4 for which fluxes from sediments could be prescribed, as a fraction of the respective downward fluxes of solid-phase iron and POM. The upward flux of Fe^{2+} from groundwater was simulated as an additional flux through the lower domain boundary.

Photosynthetic primary production was assumed to be distributed in the upper water column according to a prescribed (exponential) function of depth, and was assumed to produce POM. Oxygen was produced from that reaction for oxygentic photosynthesis, whereas anoxygenic pathways (where applicable, in lakes where light was known to reach anoxic layers) produced, respectively, FeOOH and S^0 (Table S1). The rate of particulate organic carbon mineralization (hydrolysis) was assumed to follow the power law of Katsev and Crowe (2015), which stipulates different reactivities under oxic vs. anoxic conditions. Aerobic respiration was treated as biomass-implicit (no explicit biomass pool), and was assumed to consume acetate at the maximum rate that matched that of hydrolysis. Precipitation of iron sulfides in the water column was considered to produce FeS. Pyrite formation was assumed to take place subsequently in the sediment and was not explicitly modelled.

The set of equations (8) was solved as an initial-value problem using MATLAB's *pdepe* solver. Solutions were propagated for sufficiently long times to reach steady state, typically 1000 years, with final profiles being used on output.

Additional details of model formulation In the bioenergetics model, the microbial death term was described as

$$\lambda(m_G) = \lambda_{death} - \min\left[0, k_{death}\left(\frac{rF_T \Delta G_{cat}}{P_{maint}} - 1\right)\right] \quad (9)$$

This formulation accounts for an increased rate of cell death when the obtained catabolic power falls below the minimum maintenance power requirement P_{maint}, as well as a lower, uniform death rate λ_{death} from viral lysis, etc. The maintenance power (kJ/mol_x/h) was calculated as a function of absolute temperature according to Tijhuis *et al.* (1993):

$$P_{maint} = 4.5 \exp\left[-\frac{69}{R}\left(\frac{1}{T} - \frac{1}{298}\right)\right] \quad (10)$$

Anabolism was assumed to slow down at very low concentrations of acetate (as a carbon source), which was described by a Monod-type kinetic term with the half-saturation constant $K_{ana} = 0.01$ μM.

The depth variation in mixing by turbulent eddy diffusion was simulated as described by Eq. **??** in the main text. This functional form mimics the greater intensity of mixing in the surface waters about the thermocline, and also accounts for an increase in mixing below the thermocline, which can result from a weaker density gradient there and the interactions of turbulent eddies with the bottom (Katsev *et al.* 2010).

The depth distribution of photosynthetic primary production was described as

$$R_{PP}(z) = \frac{2PP}{\sigma\sqrt{2\pi}} e^{-z^2/2\sigma^2}; \quad (11)$$

where the half-width σ was set to $H_K/2$. The integral of this function over depth equals PP.

The reactivity of POM was described as a function of ambient oxygen concentration and the age of organic material:

$$k(z, [O_2]) = f k_{ox} + (1 - f) k_{anox} \tag{12}$$

where the partitioning coefficient is $f = [O_2]/(K_{O2} + [O_2])$. The reactivities for the oxic and anoxic mineralization are (Katsev and Crowe 2015):

$$k_{ox} = 10^{-0.312} \tau^{-0.977} \tag{13}$$

$$k_{anox} = 10^{-1.1} \tau^{-0.857} \tag{14}$$

where the age τ of organic material (in years) was calculated at each depth as

$$\tau = \tau_{init} + \int_0^z \frac{dz}{v_{diff} + v_{settleG}} \tag{15}$$

The settling velocity for organic particles $v_{settleG}$ was considered in the model to be 1.5 times slower than the velocity v_{settle} for mineral particles. The effective downward velocity $v_{diff} = d\bar{z}/dt$ due to turbulent diffusion here reflects the root-mean-square displacement that in a diffusive process increases as a square root of time $\bar{z} = \sqrt{2K_z t}$; it was calculated accordingly as

$$v_{diff} = \frac{1}{\frac{z}{K_z} - (\frac{z}{2K_z})^2 \frac{dK_z}{dz}} \tag{16}$$

The flux of Fe^{2+} from sediments was assumed to be zero under oxic ($O_2 > 0.1\mu$M) or sulfidic ($H_2S > 1\mu$M) bottom conditions. Otherwise, it was calculated from the near-bottom downward flux of particulate iron F_{FeIII} according to the specified efficiency of recycling f_{rec}, up to a maximum imposed by the available flux F_{OM} of settling organic matter and the 1 : 4 stoichiometry of dissimilatory iron reduction:

$$F_{Fe-sed} = f_{rec} F_{FeIII} \min \left[1, \frac{F_{OM}}{4F_{FeIII}} \right] \tag{17}$$

Caveats and limitations The reaction-transport model does not account for the cycling of nitrate or manganese oxides, which were omitted for simplicity. These species participate in the organic matter oxidation sequence before iron oxides and sulfate and can also serve as oxidants for ferrous iron. While Mn cycling usually happens in a narrow depth interval, in a chemically stratified water column the zone of nitrate reduction can be substantial, shifting the zones of iron and sulfate reduction downward. Competitions of these pathways with denitrification, however, merit a separate study. In meromictic Lake Malawi, for example, zones of nitrate reduction and sulfate reduction overlap substantially (Li *et al.* 2018).

In evaluating the kinetic competitiveness of pathways, we did not model hydrogenotrophic pathways (Beulig *et al.* 2018), nor the forward and reverse acetogenesis. These may, in principle, influence metabolic competitiveness under some conditions. We made the choice not to include them in this study for reasons of clarity, focusing solely on the competition among the heterotrophic acetate-dependent pathways.

References

Beulig F, Røy H, Glombitza C, Jørgensen BB, Karl DM. 2018. Control on rate and pathway of anaerobic organic carbon degradation in the seabed. Proceedings of the National Academy of Sciences. 115:367–372.

González-Cabaleiro R, Ofiţeru ID, Lema JM, Rodríguez J. 2016. Microbial catabolic activities are naturally selected by metabolic energy harvest rate. The ISME Journal. 9:2630–2641.

Heijnen JJ, Van Dijken JP. 1992. In search of a thermodynamic description of biomass yields for the chemotrophic growth of microorganisms. Biotechnology and Bioengineering. 39:833–858.

Ho TY, Scranton MI, Taylor GT, Varela R, Thunell RC, Muller-Karger F. 2002. Acetate cycling in the water column of the cariaco basin: seasonal and vertical variability and implication for carbon cycling. Limnology and Oceanography. 47:1119–1128.

Jin Q, Bethke CM. 2007. The thermodynamics and kinetics of microbial metabolism. American Journal of Science. 307:643–677.

Katsev S, Crowe S. 2015. Organic carbon burial efficiencies in sediments: The power law of mineralization revisited. Geology. 43:607–610.

Katsev S, Crowe S, Mucci A, Sundby B, Nomosatryo S, Douglas Haffner G, Fowle D. 2010. Mixing and its effects on biogeochemistry in the persistently stratified, deep, tropical Lake Matano, Indonesia. Limnology and Oceanography. 55:763–776.

Katsev S, Halevy I. 2025. Combined thermodynamic-kinetic-competitive controls on anaerobic respiration pathways and the chemistry of natural waters. Earth ArXiV doi:10.31223/X53X79.
.

Lee C, Peterson ML, Wakeham SG, Armstrong RA, Cochran JK, Miquel JC, Fowler SW, Hirschberg D, Beck A, Xue J. 2009. Particulate organic matter and ballast fluxes measured using time-series and settling velocity sediment traps in the northwestern Mediterranean Sea. Deep-Sea Research Part II: Topical Studies in Oceanography. 56:1420–1436.

Li J, Brown ET, Crowe SA, Katsev S. 2018. Sediment geochemistry and contributions to carbon and nutrient cycling in a deep meromictic tropical lake: Lake Malawi (East Africa). Journal of Great Lakes Research. 44:1221–1234.

Roden EE, Wetzel R. 2003. Competition between fe (iii)-reducing and methanogenic bacteria for acetate in iron-rich freshwater sediments. Microbial Ecology. 45:252–258.

Smeaton CM, Van Cappellen P. 2018. Gibbs Energy Dynamic Yield Method (GEDYM): Predicting microbial growth yields under energy-limiting conditions. Geochimica et Cosmochimica Acta. 241:1–16.

Stams A, Plugge CM, De Bok FA, Van Houten B, Lens P, Dijkman H, Weijma J. 2005. Metabolic interactions in methanogenic and sulfate-reducing bioreactors. Water Science and Technology. 52:13–20.

Tijhuis L, Van Loosdrecht MC, Heijnen JJ. 1993. A thermodynamically based correlation for maintenance gibbs energy requirements in aerobic and anaerobic chemotrophic growth. Biotechnology and Bioengineering. 42:509–519.

Table. S3 Lake-independent model parameters

Parameter	Value	Unit	Comment
τ_{init}	0.1	y	initial age of organic matter (Katsev and Crowe 2015)
K_{Ac}	5/15.5/12	μM	acetate half-saturation constant for SR/IR/MG (Ho *et al.* 2002; Roden and Wetzel 2003; Stams *et al.* 2005)
v_{settle}	700	m/y	settling velocity (Lee *et al.* 2009)
			Parameters below are the same as in Katsev and Halevy (2025)
K_{O2}	2	μM	oxygen half-saturation constant
K_{O2}^{inh}	1	μM	oxygen half-inhibition constant for anaerobes
K_{SO4}	5	μM	sulfate half-saturation constant
K_{CH4}	30	μM	methane half-saturation constant for AOM
K_{FeIII}	1000 /10	μmol/L	goethite / nanophase Fe(III) half-saturation constant
k_{HSox}	1	μM^{-1} y^{-1}	rate constant for sulfide oxidation by oxygen
k_{Feox}	1	μM^{-1} y^{-1}	rate constant for Fe^{2+} oxidation by oxygen
$k_{HSFeIII}$	0.2/4	μM^{-1} y^{-1}	rate constant for sulfide oxidation by goethite/nanophase Fe(III)
k_{FeS}	250	μM y^{-1}	rate constant for iron sulfide mineral precipitation
k_{CH4ox}	1	μM^{-1} y^{-1}	rate constant for methane oxidation by oxygen
k_{disp}	0.02	y^{-1}	rate constant for disproportionation of S^0
K_{FeS}	2.5×10^{-3}	M	equilibrium constant for FeS precipitation
ΔG_{ATP}	30	kJ/mol	cost of producing ATP
χ	8,6,2	-	stoichiometric number for IR/SR/MG
m	1.25/1/0.25	-	stoichiometric number for IR/SR/MG
V_{max}	1.0	$mol_{rx}/mol_x/h$	microbial kinetics for SR
	0.1/0.3		microbial kinetics for IR (bulk/nanophase)
	1.0		microbial kinetics for MG
	0.04		microbial kinetics for S-AOM
	0.02/0.04		microbial kinetics for Fe-AOM (bulk/nanophase)
λ_{death}	2×10^{-4}	h^{-1}	death rate constant
k_{death}	2×10^{-3}	h^{-1}	death rate from energy starvation
$[HCO_3^-]$	3	mM	ambient concentration
$[NH_4^+]$	10	μM	ambient concentration of nutrient for anabolism
$[H_2]$	0.1	μM	ambient concentration

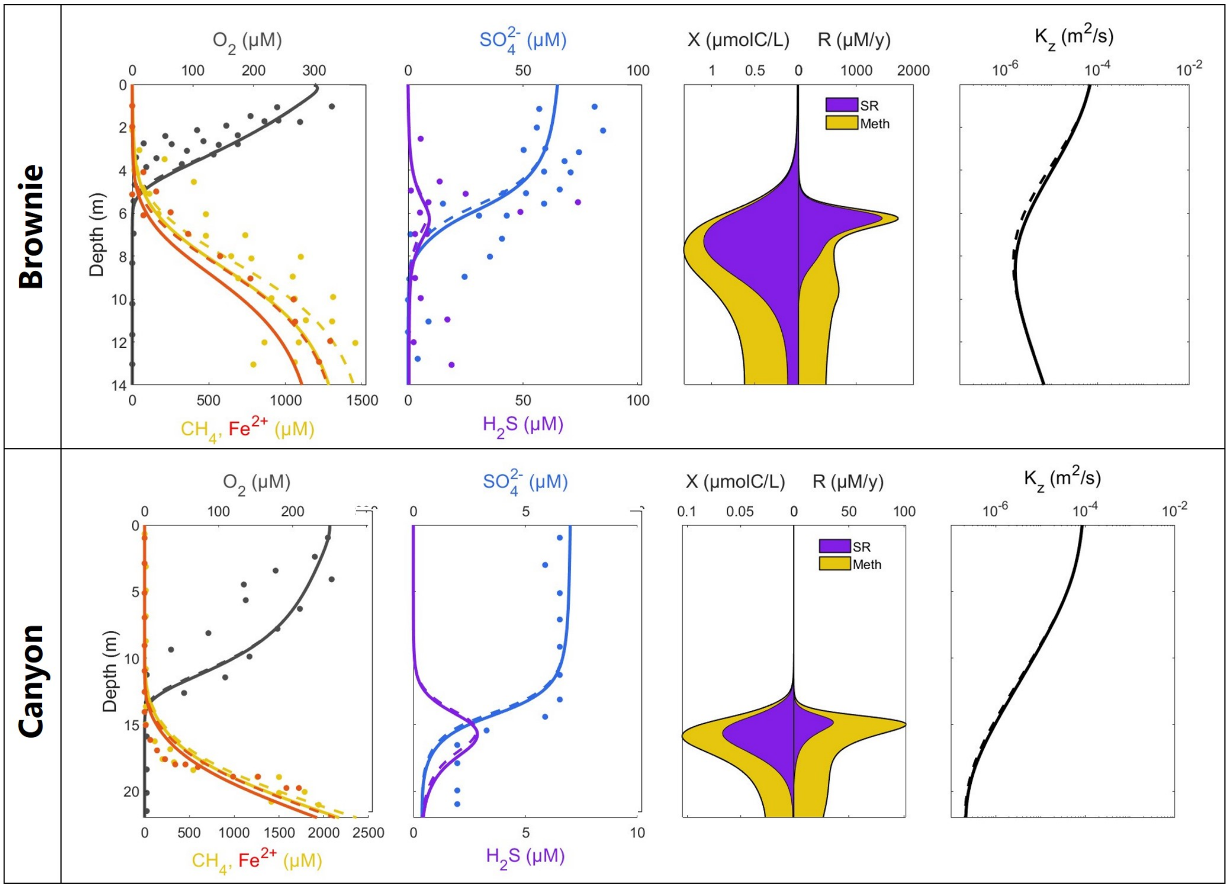

Fig. S1 Model simulations for Brownie Lake and Canyon Lake using different thermocline half-width values. Solid line simulations have $h_K = 1.32$ m for Brownie and $h_K = 2.31$ m for Canyon. Dashed lines represent chemical profiles for each lake in Figure 2.
